# Improved patient-reported outcomes after open preperitoneal inguinal hernia repair compared to anterior Lichtenstein repair: 10-year ACHQC analysis

**DOI:** 10.1007/s10029-023-02852-6

**Published:** 2023-08-08

**Authors:** Divyansh Agarwal, Tina Bharani, Nora Fullington, Lauren Ott, Molly Olson, Benjamin Poulose, Jeremy Warren, Michael Reinhorn

**Affiliations:** 1 Department of Surgery, Massachusetts General Hospital, 55 Fruit St., GRB 425, Boston, MA 02114 USA; 2 Brigham and Women’s Hospital, Department of Surgery, Boston, MA USA; 3 Boston Hernia and Pilonidal Center, 20 Walnut Street, Suite 100, Wellesley, MA 02481 USA; 4 Mass General Brigham - Newton Wellesley Hospital, Newton, MA USA; 5 Tufts University School of Medicine, Boston, MA USA; 6 Department of Population Health Sciences, Weill Cornell Medicine, New York, NY USA; 7 Center for Abdominal Core Health, Department of Surgery, The Ohio State University Wexner Medical Center, Columbus, OH USA; 8 Department of Surgery, Division of Minimal Access, and Bariatric Surgery, Prisma Health Upstate, 701 Grove Rd, ST 3, Greenville, SC 29605 USA

**Keywords:** Lichtenstein, Inguinal hernia, TREPP/OPP, Quality-of-life, Preperitoneal repair

## Abstract

**Introduction:**

The Lichtenstein repair has been synonymous with “open” inguinal hernia repair (IHR) for 40 years. However, international guidelines have suggested that posterior mesh placement results in advantageous biomechanics and reduced risk of nerve-related chronic pain. Additionally, the use of local anesthetics has been shown to reduce postoperative pain and complication risks. An open transrectus preperitoneal/open preperitoneal (TREPP/OPP) repair combines posterior mesh placement with the use of local anesthetic and as such could be the ideal repair for primary inguinal hernia. Using the Abdominal Core Health Quality Collaborative (ACHQC) registry, we compared open anterior mesh with open posterior mesh repairs.

**Methods:**

We performed a propensity score matched analysis of patients undergoing open IHR between 2012 and 2022 in the ACHQC. After 1:1 optimal matching, both the TREPP/OPP and Lichtenstein cohorts were balanced with 451 participants in each group. Outcomes included patient-reported quality of life (QoL), hernia recurrence, and postoperative opioid use.

**Results:**

Improvement was seen after TREPP/OPP in EuraHS QoL score at 30 days (OR 0.558 [0.408, 0.761]; *p* = 0.001), and the difference persisted at 1 year (OR 0.588 [0.346, 0.994]; *p* = 0.047). Patient-reported opioid use at 30-day follow-up was significantly lower in the TREPP/OPP cohort (OR 0.31 [0.20, 0.48]; *p* < 0.001). 30-day frequency of surgical-site occurrences was significantly higher in the Lichtenstein repair cohort (OR 0.22 [0.06–0.61]; *p* = 0.007). There were no statistically significant differences in hernia recurrence risk at 1 year, or rates of postoperative bleeding, peripheral nerve injury, DVTs, or UTIs.

**Conclusion:**

Our analysis demonstrates a benefit of posterior mesh placement (TREPP/OPP) over anterior mesh placement (Lichtenstein) in open inguinal hernia repair in patient-reported QoL and reduced opioid use.

## Introduction

Inguinal hernia repair (IHR) remains one of the most common surgical interventions worldwide, with over 20 million IHR procedures performed annually [1]. While watchful waiting is a safe option for small hernias or those with minimal symptoms, two-thirds of patients progress to require surgical repair within 10 years of their initial diagnosis [2, 3]. Surgery remains the definitive repair option for all hernias [4]. Over 100 IHR techniques have been described in the literature, however, which approach is the most optimal for long-term patient outcomes remains an area of investigation [5]. The ideal IHR must be associated with minimal complications, safe and swift recovery, low recurrence rate, low risk of chronic pain, and be both cost-effective and reproducible so as to enable widespread global adoption [1].

Anterior mesh IHR via the Lichtenstein approach remains the most common technique with reported low recurrence and complication rate [6]. Despite wide adoption of this technique, many patients are harmed by this approach and experience debilitating chronic postoperative inguinal pain. In the US alone, an estimated 3% of 800,000 patients are disabled every year by the most common hernia repair performed in the US [7–10]. However, minimally invasive laparoscopic repair including transabdominal preperitoneal (TAPP) repair and totally extra-peritoneal repair (TEP) have shown reduction in rates of post-operative complications, wound infections, chronic pain, comparable recurrence rates, and quicker return to work compared to Lichtenstein repair [6, 11]. Reduced risk of chronic pain in laparoscopic procedures has been associated with mesh placement in the preperitoneal space, which circumvents the dissection and manipulation of nerves in the inguinal canal required in anterior mesh placement [12, 13]. Mesh placement in the preperitoneal space provides wide overlap of the myopectineal orifice of Fruchaud (MPO). Biomechanically, the intra-abdominal pressure pushes the mesh against the abdominal wall, keeping it positioned, rather than pushing it away, a phenomenon termed as the upstream principle [14]. In contrast, anterior mesh placement in the Lichtenstein approach does not benefit from this principle and hence requires more aggressive fixation of the mesh to avoid abdominal pressure pushing it away from the abdominal defect [14], which in turn increases the risk of nerve entrapment and the likelihood of chronic pain [15]. International guidelines have concurred that posterior mesh placement results in less acute postoperative pain, less chronic pain and faster recovery [1, 16, 17]. Similarly, a meta-analysis comparing pre-peritoneal mesh repair to anterior Lichtenstein approach showed significantly reduced risk of chronic groin pain with preperitoneal approach with no significant difference in hernia recurrence [16].

The open transrectus preperitoneal/open preperitoneal (TREPP/OPP) approach provides the benefits of mesh placement in the preperitoneal space while minimizing the cost and resources necessary for a laparoscopic or robotic IHR approach. In addition, TREPP/OPP can be performed under local anesthesia with sedation, in contrast to laparoscopic repair which requires general anesthesia to facilitate muscle relaxation for pneumoperitoneum and mesh placement [18].

In our analysis, we sought to compare a pure anterior approach with a pure posterior approach and to exclude repairs that violate both the anterior and posterior planes, or repairs that require laparoscopic equipment and general anesthesia with muscle paralysis [16]. We hypothesized that in similar groups, patients who undergo an open posterior inguinal hernia repair will have significantly improved QoL compared to patients who undergo a traditional open anterior mesh repair.

## Methods

### Data collection

The Abdominal Core Health Quality Collaborative (ACHQC) is a national US-based registry that collects short- and long-term hernia-specific data, including patient-reported outcomes related to hernia repairs with the goal of improving surgical quality and value [23]. We utilized data collected in the ACHQC to compare anterior (Lichtenstein) versus open posterior (TREPP) mesh repairs of unilateral inguinal hernia. This study was approved by the Institutional Review Board at Prisma Health Upstate. Informed consent was obtained from all patients prior to collecting their clinical data for the ACHQC.

Between August 2012 and July 2022, 28,389 patients underwent inguinal hernia repair. Numerous repairs have been described to obtain ideal preperitoneal mesh placement, including TEP, TAPP, rTAPP, rTEP, TRIPP, modified Kugel, and STOPPA. Patients who underwent bilateral inguinal hernia repair, minimally invasive (laparoscopic and robotic) approaches, trans-inguinal posterior approaches, combined inguinal and ventral hernia repair, or repair of multi recurrent (> 1 recurrence) inguinal hernias were excluded. Among the 3,555 patients who met the inclusion criteria, 1,050 patients underwent TREPP and 2,505 underwent a Lichtenstein repair. We then matched 451 patients in the Lichtenstein IHR cohort with 451 patients in the TREPP group for our analysis. Table 1 highlights the factors that were matched between the two cohorts.

**Table 1:** Standardized mean differences (SMDs) in baseline characteristics in the Lichtenstein/anterior and OPP/TREPP IHR cohorts after propensity score matching

	Lichtenstein	TREPP	SMD
*N*	451	451	
Age capped at 90 (mean (SD))	60.87 (14.19)	60.53 (14.53)	0.024
Gender = Male (%)	424 (94.0)	424 (94.0)	< 0.001
Race/ethnicity = Other (%)	33 ( 7.3)	34 ( 7.5)	0.008
BMI (mean (SD))	25.96 (4.01)	26.02 (4.04)	0.017
Insurance (%)			0.073
Private	290 (64.3)	284 (63.0)	
Medicare	148 (32.8)	148 (32.8)	
Other/Unknown	13 ( 2.9)	19 ( 4.2)	
ASA class (%)			0.079
1	103 (22.8)	105 (23.3)	
2	285 (63.2)	271 (60.1)	
3	63 (14.0)	75 (16.6)	
Hypertension = Yes (%)	136 (30.2)	139 (30.8)	0.014
Diabetes Mellitus = Yes (%)	24 ( 5.3)	20 ( 4.4)	0.041
Chronic Obstructive Pulmonary Disease = Yes (%)	2 ( 0.4)	3 ( 0.7)	0.03
Anti-platelet medications = Yes (%)	53 (11.8)	55 (12.2)	0.014
Anti-coagulation medications = Yes (%)	5 ( 1.1)	9 ( 2.0)	0.072
Smoker within one year = Yes (%)	18 ( 4.0)	21 ( 4.7)	0.033
Enlarging hernia = Yes (%)	28 ( 6.2)	25 ( 5.5)	0.028
Painful bulge = Yes (%)	441 (97.8)	437 (96.9)	0.055
Recurrent hernia = Yes (%)	38 ( 8.4)	35 ( 7.8)	0.024
Prior mesh = Yes (%)	15 ( 3.3)	15 ( 3.3)	< 0.001
Medial type hernia size (%)			0.099
No Hernia	281 (62.4)	300 (66.5)	
I (< 1.5 cm or < 1 fingertip)	20 ( 4.4)	22 ( 4.9)	
II (1.5-3 cm or 1–2 fingertips)	114 (25.3)	97 (21.5)	
III (> 3 cm or > 2 fingertips)	35 ( 7.8)	32 ( 7.1)	
Lateral type hernia size (%)			0.07
No Hernia	132 (29.3)	120 (26.7)	
I (< 1.5 cm or < 1 fingertip)	62 (13.7)	67 (14.9)	
II (1.5-3 cm or 1–2 fingertips)	223 (49.4)	224 (49.8)	
III (> 3 cm or > 2 fingertips)	34 ( 7.5)	39 ( 8.7)	
Scrotal component = Yes (%)	20 ( 4.4)	26 ( 5.8)	0.061
History of other substance use = Yes (%)	160 (51.8)	241 (54.9)	0.063
Patient or surgeon reported opioid use in last 30-days at baseline = 1 or more opioids (%)	2 ( 0.6)	5 ( 1.1)	0.058
Any behavioral health hx = Yes (%)	15 ( 4.9)	30 ( 6.8)	0.084
EuraHS overall score at baseline (mean (SD))	26.24 (18.61)	27.60 (19.72)	0.071
EuraHS score for baseline pain (mean (SD))	6.93 (6.29)	7.42 (6.26)	0.078
EuraHS score for baseline esthetical (mean (SD))	7.30 (5.22)	7.34 (5.23)	0.008
EuraHS score for baseline restriction (mean (SD))	11.99 (10.94)	12.88 (11.91)	0.078

The *N* is italicized to suggest that it is referring to the number of subjects

### Characterization of open preperitoneal IHR

**Fig. 1:**
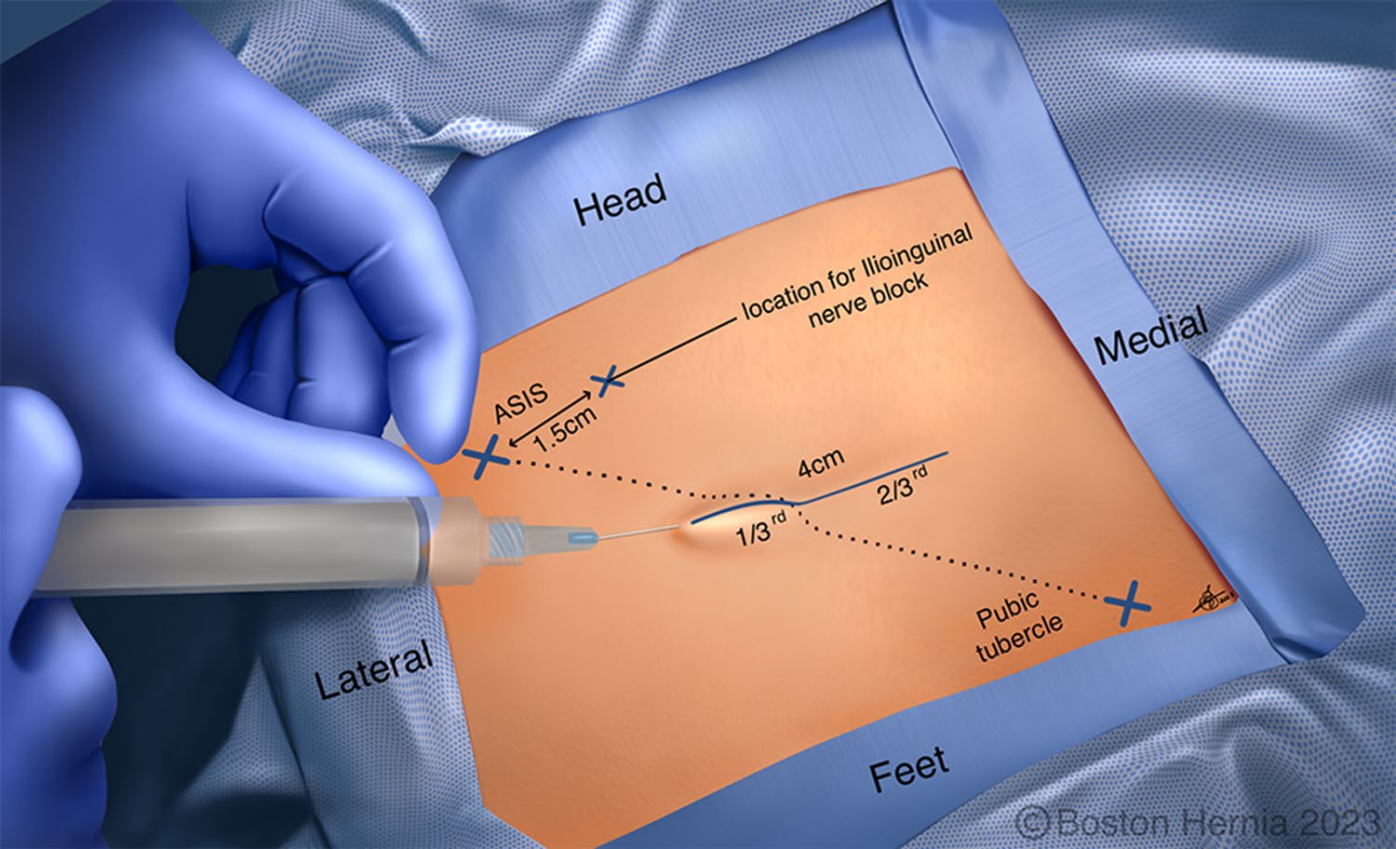
Incision is approximately 4 cm long, and 2/3rd medial to the midpoint between ASIS and the pubic tubercle

**Fig. 2:**
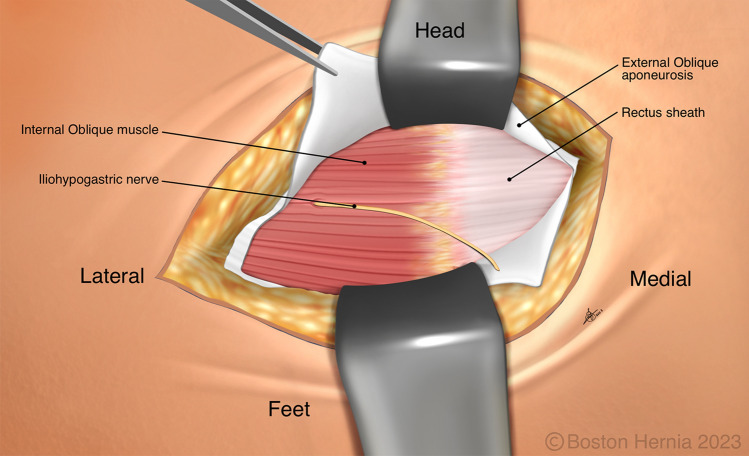
The external oblique has been opened, and the iliohypogastric nerve is typically seen along the internal oblique and rectus sheath

**Fig. 3:**
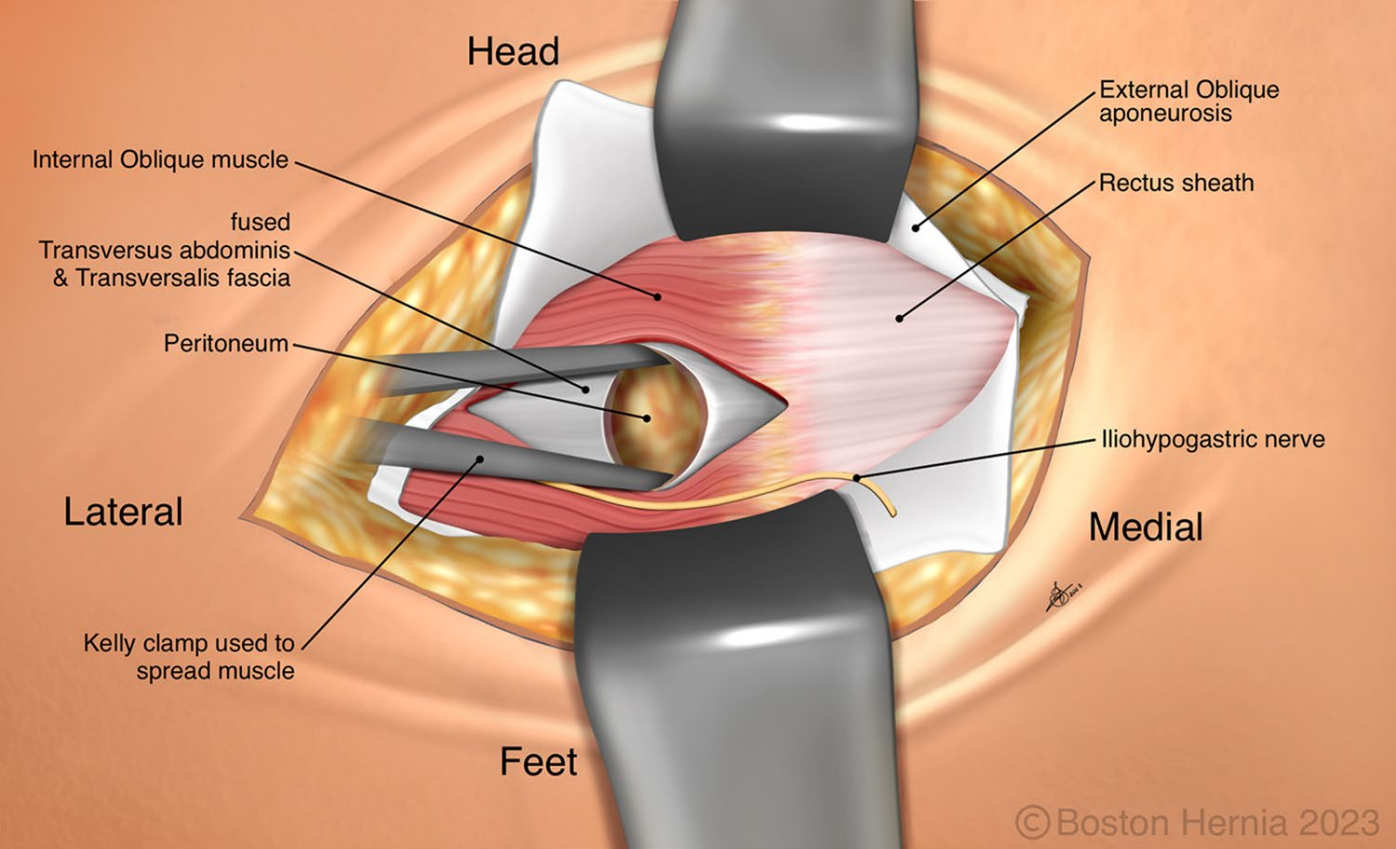
The internal obliques are separated at the lateral edge of the rectus sheath, typically above the iliohypogastric nerve

**Fig. 4:**
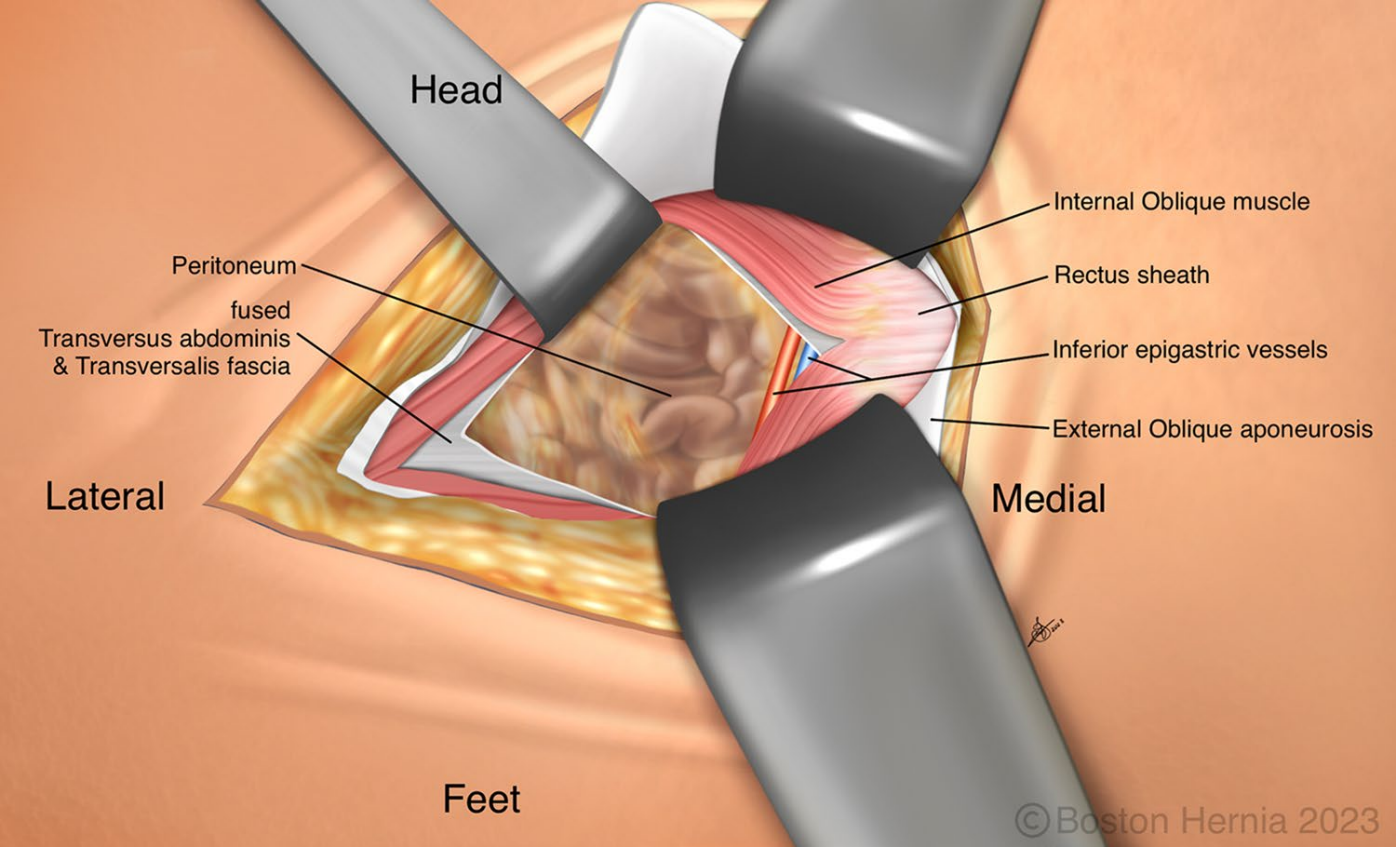
The aponeurosis of the transversus abdominis, which are sometimes fused with the transversalis fascia, must be divided to gain access to the preperitoneal plane

**Fig. 5:**
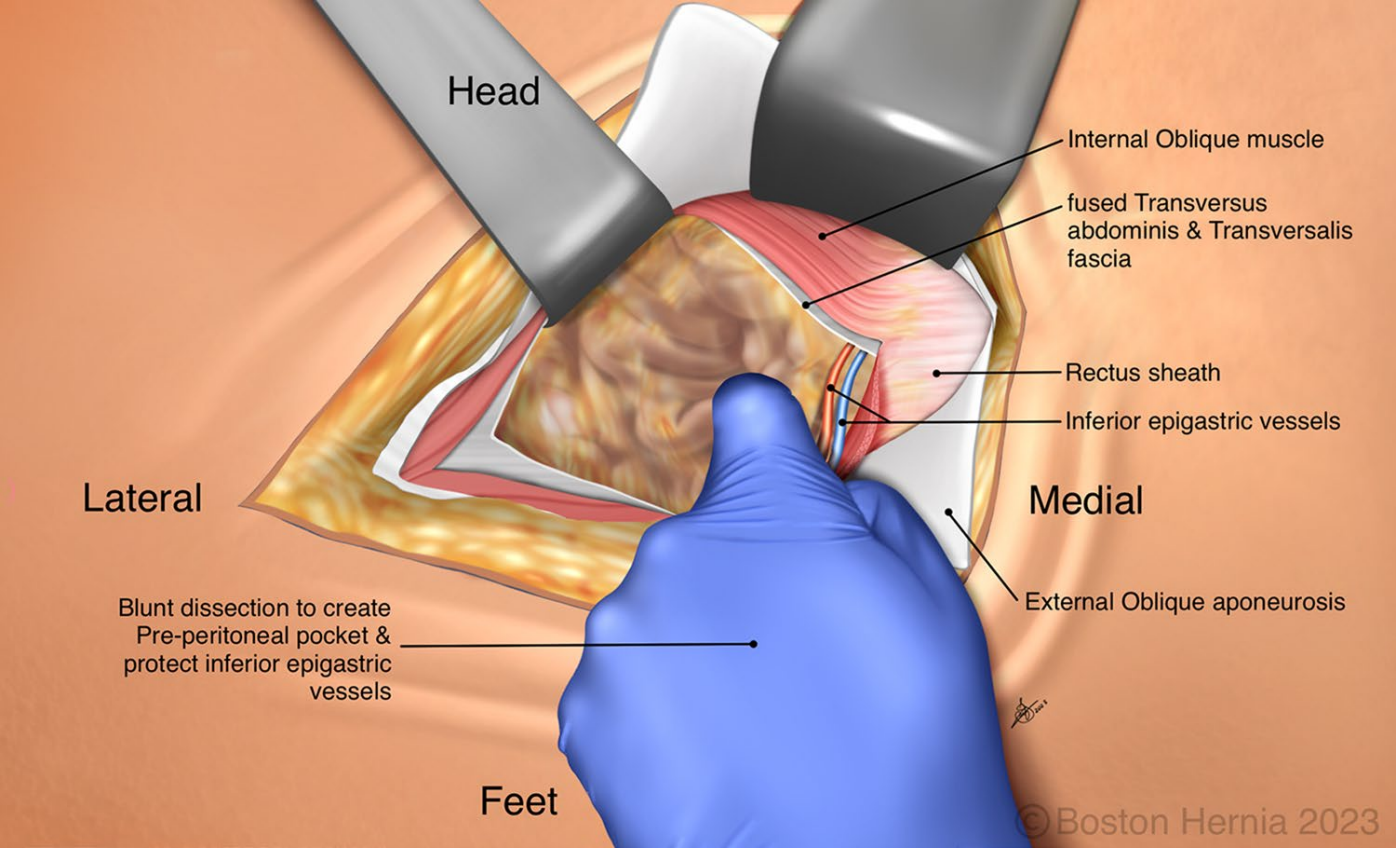
Care must be taken to identify and to protect the inferior epigastric vessels, by feeling or seeing them, and then retracting them medially with the rectus which is superficial. Blunt finger dissection can then be used to create a preperitoneal pocket, similar to a balloon dissector in a TEP or laparoscopic instruments in a TAPP. The peritoneum is rarely ever entered

**Fig. 6:**
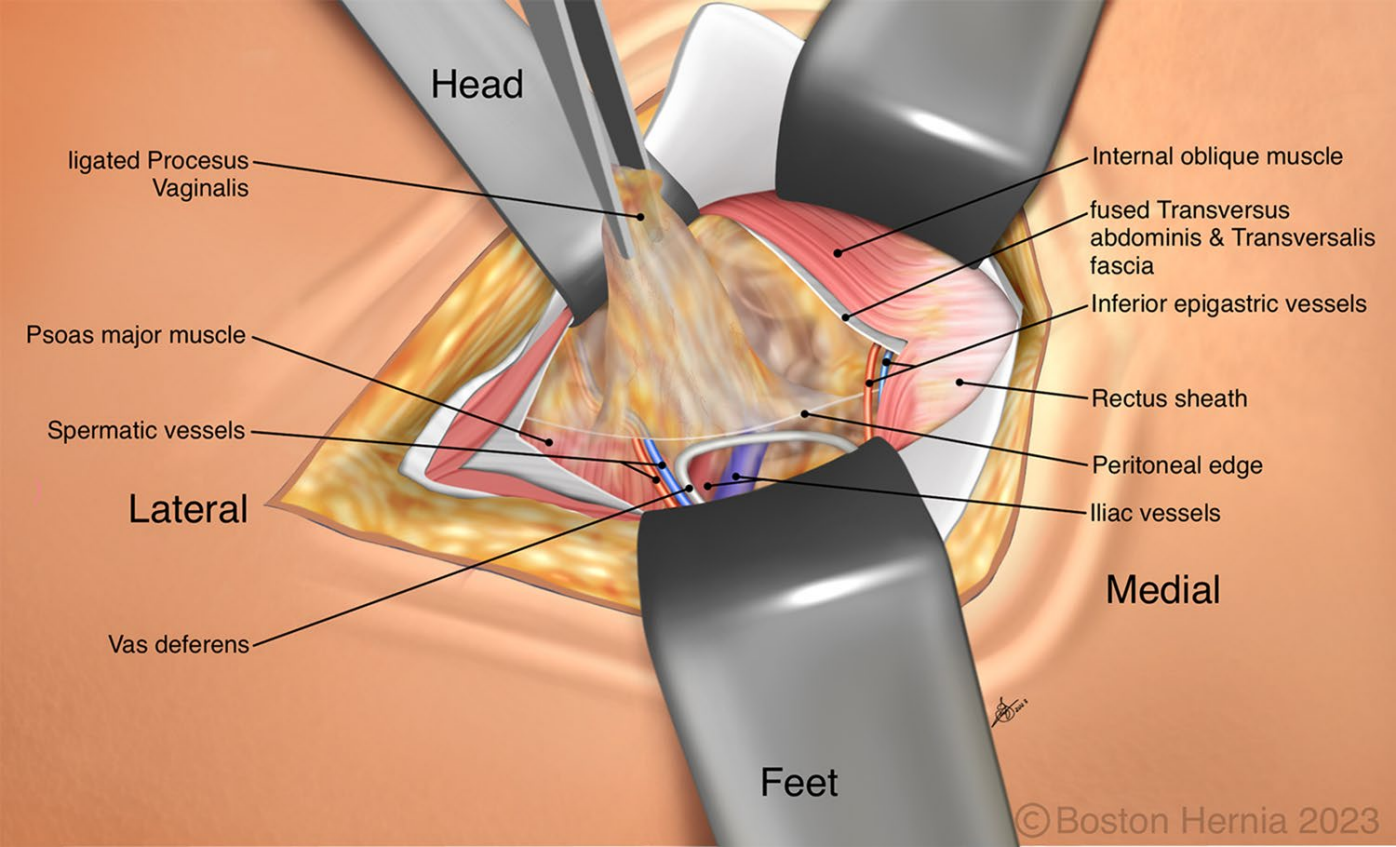
Dissection of the peritoneal sac and preperitoneal fat is straightforward as the peritoneum is immediately accessed, and is superficial to the spermatic vessels. A high ligation is performed for a large inguinal or scrotal hernia

**Fig. 7:**
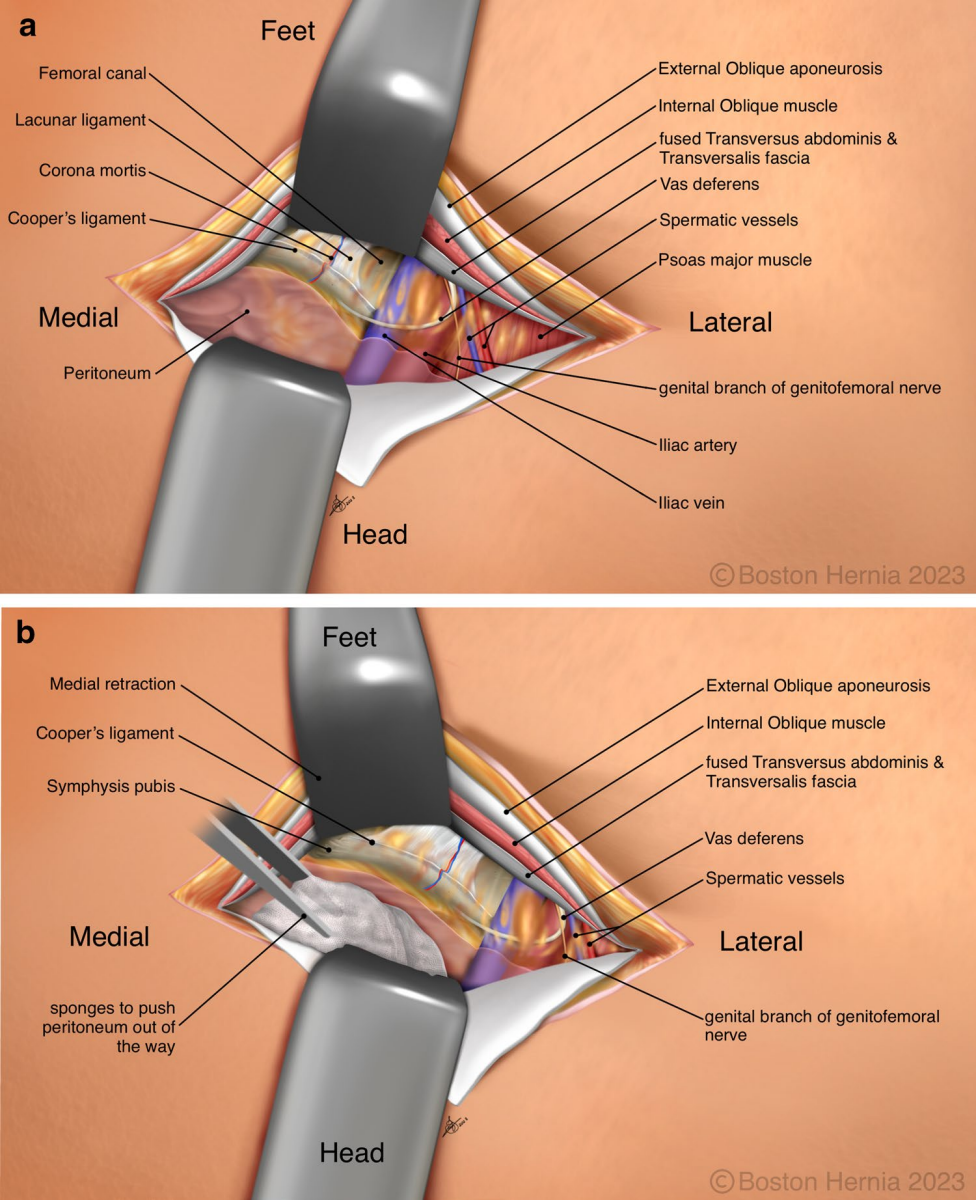
**a** Surgeons view looking down toward the pelvis demonstrating the anterior abdominal wall structures. Confirmation of peritoneal dissection is done by observing the course of the vas into the deep pelvis. This view is aided using a surgical headlight. **b** The medial dissection is performed with minimal electrocautery as this is an avascular plane. Dissection is concluded when the pubic symphysis is cleared and tissue is cleared 2 cm below and deep to the pubic bone. The iliac vein is clearly seen in all but the morbidly obese patients

**Fig. 8:**
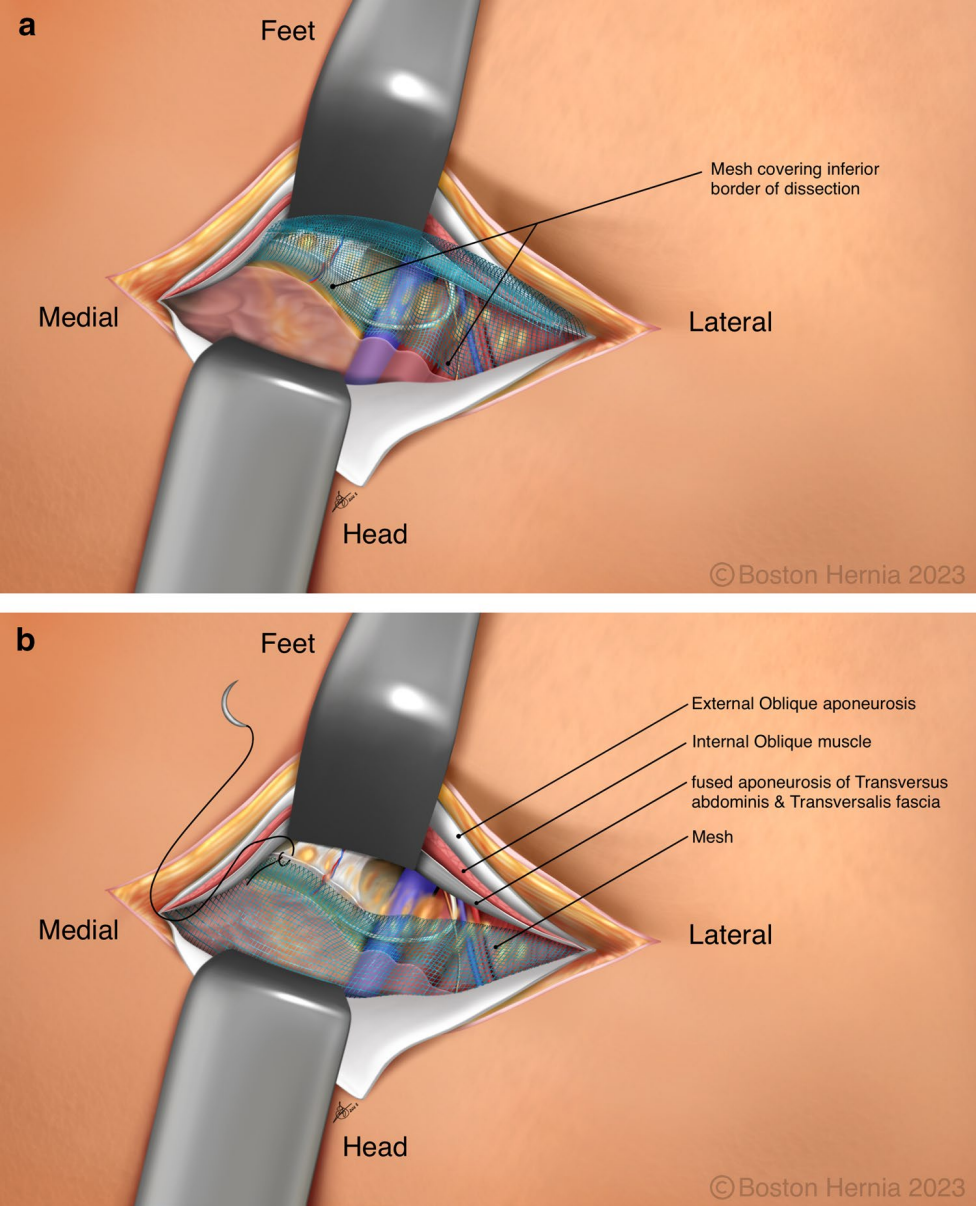
**a** The mesh is inserted and placed carefully covering the entire myopectineal orifice of Fruchaud, and the peritoneum is pulled up to ensure no movement or curling of the mesh. **b** The anterior portion of the mesh is flipped down to cover the peritoneum and the bladder. It is then sutured to the Cooper’s ligament under direct visualization

**Fig. 9:**
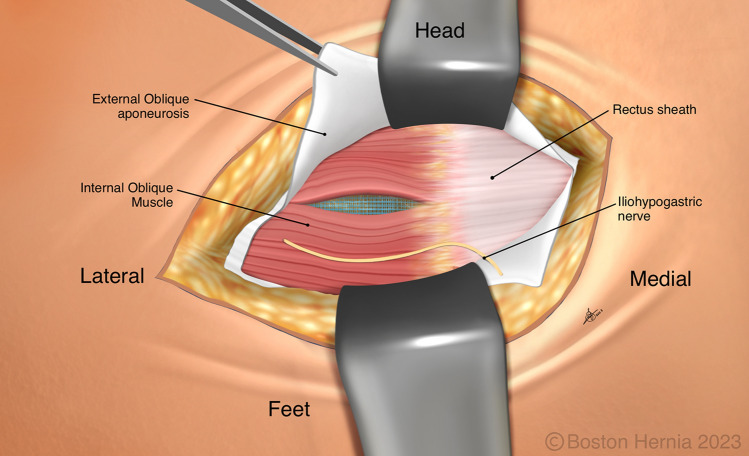
The internal obliques are allowed to come together naturally over the mesh to avoid suturing near the iliohypogastric nerve

**Fig. 10:**
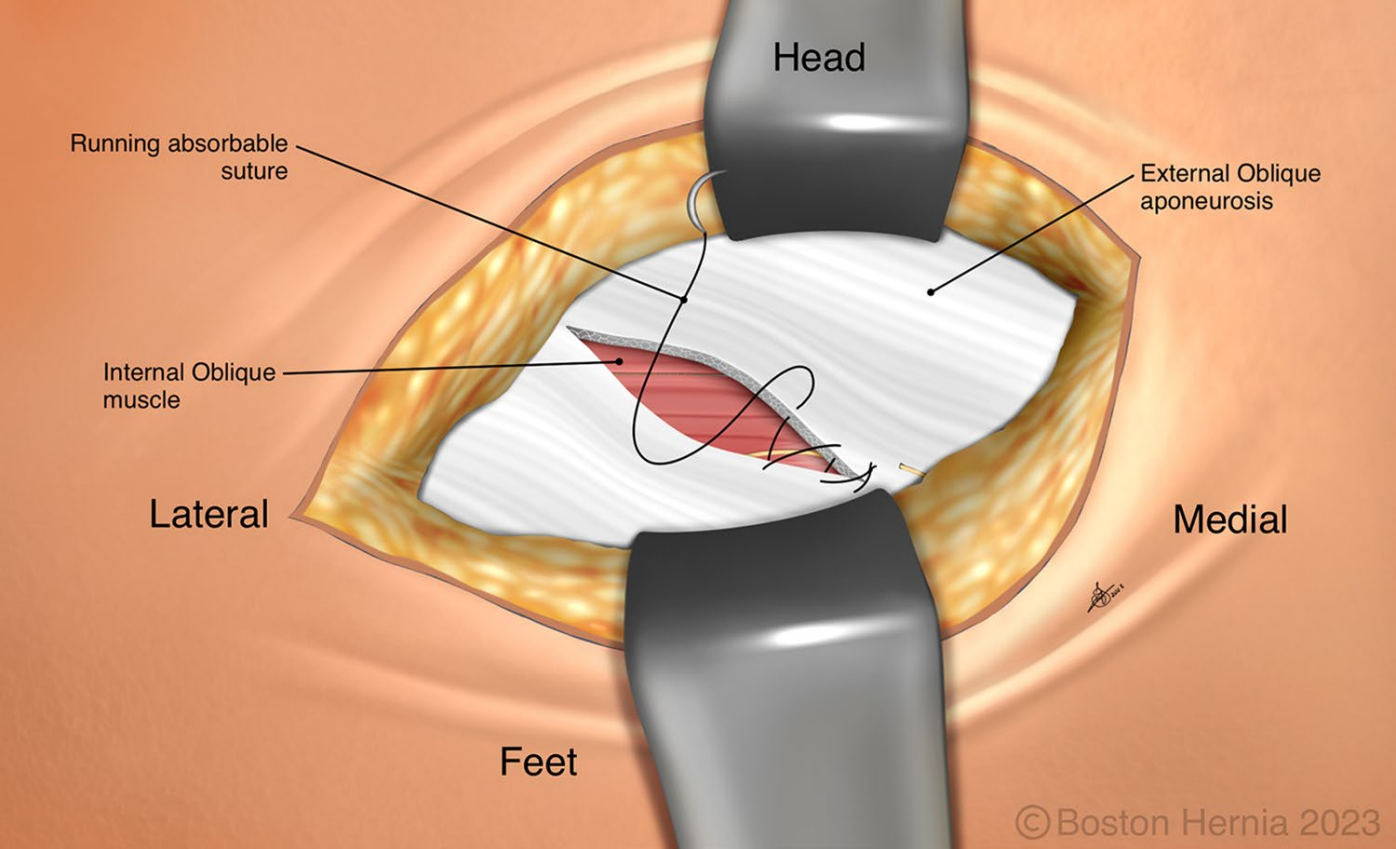
The external oblique aponeurosis is closed with a running absorbable suture, taking care not to entrap the iliohypogastric nerve

In the ACHQC, open posterior mesh approaches that do not violate the anterior plane were grouped under TREPP. These include OPP, TREPP, and Kugel. As previously described [14, 18–22, 24], these approaches involve a lower abdominal incision and opening of the external oblique aponeurosis superior to the inguinal canal. This dissection avoids the inter-parietal plane between the external and internal obliques where anterior repair is typically performed, thus minimizing scarring in the inguinal canal and allowing unobstructed anterior repair in the event of recurrence requiring future anterior repair. In medial defects, excess transversalis fascia is inverted and sutured to Cooper's ligament as well [25, 26]. The procedure is typically performed in the following steps [27]:

1. Incision at the midpoint between the anterior superior iliac spine (ASIS) and the pubic tubercle (Fig. 1),
2. Exposure and identification of internal oblique, rectus sheath, and Iliohypogastric nerve (Fig. 2),
3. Divide the rectus sheath and retract the rectus, versus splitting the internal oblique muscles (Fig. 3),
4. Divide the aponeurosis of transversus abdominis and transversalis fascia to gain access to the preperitoneal space (Fig. 4),
5. Identify the inferior epigastric vessels and create a preperitoneal pocket using blunt dissection (Fig. 5),
6. Dissect the indirect component of the hernia past the bifurcation of the vas deferens and the spermatic vessels (Fig. 6),
7. Confirm that the dissection of the peritoneum is complete (Fig. 7a),
8. Perform medial dissection of the direct and femoral spaces to the pubic symphysis and below Cooper’s ligament (Fig. 7b),
9. Insert the mesh, verify appropriate placement, and inspect the peritoneum (Fig. 8a),
10. Mesh fixation using simple interrupted sutures (Fig. 8b),
11. Close rectus sheath versus allowing the internal oblique muscles to spring back in place, based on the operative approach taken in Step 3 (Fig. 9),
12. Close the external oblique aponeurosis, taking care to avoid injury to the iliohypogastric nerve (Fig. 10).

Note that Figs. 1–6 demonstrate a right inguinal hernia undergoing TREPP from the right side of the operating surgeon. Figures 7–10 demonstrate the right inguinal hernia repair from the left side of the operating surgeon, as the surgeon looks toward the pelvis.

### Outcomes

Patient-reported QoL metrics have been described as the most important outcome measure after an IHR [15]. We compared patient-reported QoL as well as longitudinal clinical outcomes between the TREPP/OPP and an anterior/Lichtenstein IHR approach using the ACHQC registry. Data collected include patient demographics and comorbidity, surgical details, clinical outcomes, and patient-reported outcomes (PRO) before, during, and after unilateral IHR procedures, as described previously [28]. The primary outcome is patient-reported quality of life using the EuraHS scores at 30-day, 6-month, and 1-year after surgery. The EuraHS is a validated quality of life measurement tool for inguinal hernia. The tool assesses pain (range 0–30), restriction of activity (range 0–40), and cosmetic discomfort (range 0–20) due to the hernia or from surgery [29] with total scores ranging from 0 to 90. A lower score signifies an improved QOL. We also assessed patient-reported opioid use at 30-day follow-up.

Secondary outcomes include perioperative complications, surgical-site occurrence or infection, and composite hernia recurrence. Of note, surgical-site infection was defined as a deep incisional, superficial incisional, or organ space infection, whereas surgical-site occurrence was defined as wound cellulitis, fascial disruption, wound drainage, seroma, hematoma, contaminated or infected mesh, entero-cutaneous fistula formation, or skin or soft tissue ischemia. Clinical or radiographic recurrence is recorded by the clinician at any point after surgery. Patient surveys are completed at 30 days, 6 months, and then once per year after surgery. Composite recurrence is defined by the Hernia Recurrence Inventory which includes physical exam or radiographic imaging at any point post-operatively or a patient-reported bulge at the site of the hernia at the 1-year time point or beyond after an IHR.

### Statistical methods

Patient-level, hernia, and operative characteristics were compared between individuals who received TREPP and a Lichtenstein IHR. Pearson’s chi-squared and Wilcoxon rank-sum tests were used to conduct bivariate tests comparing categorical and continuous covariates, respectively. Time-to-recurrence was examined using Kaplan–Meier recurrence-free estimation and log-rank test to compare recurrence curves between operative approaches. An advantage of evaluating recurrence as time-to-event is the ability to use all information available to compute 1-year recurrence probability, including endpoint and censoring information, and to account for varying length of follow-up. Although the time it might take after an IHR for the hernia to recur is not possible to predict definitively in the clinical setting, the estimate of recurrence-free probability using the Kaplan–Meier analysis is an unbiased representation of the true time-to-event data. Additional pairwise analysis was performed to detect differences in TREPP and the Lichtenstein IHR technique.

To minimize the effects of selection bias and systematic differences in baseline covariates, we created a propensity score matched cohort. A logistic regression model was used to estimate the propensity score for operative approach conditional on covariates identified a priori. Covariates included in the propensity score model were age, gender, race, BMI, insurance status, ASA class, comorbidities, indication for surgery (enlarging hernia, painful bulge, recurrent hernia), prior pelvic operation, prior mesh, hernia size, scrotal component, history of substance use, history of opioid use, behavioral health history, and EuraHS quality of life score measured at baseline. A 1:1 nearest-neighbor matching algorithm with a caliper of 0.2 was used to match TREPP with Lichtenstein IHRs [30, 31]. Balance was assessed by examining the standardized mean differences (SMD) of baseline covariates where a SMD < 0.1 was considered good balance. Odds ratios (OR) and their 95% confidence intervals (CI) were estimated using logistic, proportional odds, or Cox proportional hazards models for binary, patient-reported, and time-to-event outcomes, respectively. To assess the difference in EuraHS quality of life scores between surgical approaches for populations with the same baseline score, we adjust for baseline scores in a proportional odds regression model.

## Results

### Baseline characteristics of patients

Between August 2012 and 2022, 28,389 patients underwent an inguinal hernia repair as part of the ACHQC. Among these, 3555 individuals met the inclusion criteria (see Methods). The TREPP approach was used to repair the hernia in 1,050 patients, whereas a Lichtenstein repair was performed in 2505 individuals. To account for confounding covariates, we used 1:1 propensity score matching (PSM) using the nearest-neighbor matching algorithm. After PSM, both the TREPP and Lichtenstein cohorts were balanced with 451 participants in each group. There were no significant differences between the adjusted groups based on age, gender, race/ethnicity, ASA class, or medical comorbidities (Table 1).

### Primary outcomes

#### Patient-report quality of life

Postoperative QoL was the primary outcome we examined in this work. In the matched analysis, after accounting for baseline scores, there was a significantly better (lower) EuraHS QoL score in TREPP compared to Lichtenstein at the 30-day (Median (IQR) 8.0 (2.0–18.0) vs 15.0 (4.0–29.0); OR 0.558 [0.408, 0.761]; *p* = 0.001) time point (Table 2). This difference was persistent even at 1-year postoperatively (1.0 (0–4.0) vs 2.0 (0.0–7.6); OR 0.588 [0.346, 0.994]; *p* = 0.047). Additionally, domain-specific sub-analysis performed post hoc revealed lower pain and restriction domain scores after TREPP repair at 30 days, but not at 180 days, in comparing the TREPP cohort to the matched Lichtenstein repair cohort (Table 3). No QoL differences were evident at any time point in the esthetic domain scores between the two groups.

**Table 2:** Overall EuraHS and opioid use patient-reported outcomes, after adjusting for preoperative opioid use

Outcome	*N*	Lichtenstein	TREPP	*P*-value	OR	95% CI
		(*N* = 451)	(*N* = 451)			
EuraHS QoL score from baseline survey (score 0–90)	363			0.589	1.104	(0.772, 1.579)
*N*		162	201			
Median (interquartile range)		21.00 (11.00–40.78)	24.00 (10.00–41.33)			
Range		0.00–82.00	0.00–82.00			
Mean ± SD		26.24 ± 18.61	27.60 ± 19.72			
EuraHS QoL score from 30-day survey (score 0–90)	493			< 0.001	0.558	(0.408, 0.761)
*N*		235	258			
Median (interquartile range)		15.00 (4.00–29.00)	8.00 (2.00–18.00)			
Range		0.00–65.00	0.00–56.00			
Mean ± SD		18.00 ± 16.25	12.56 ± 13.60			
EuraHS QoL score from 6-month survey (score 0–90)	293			0.256	0.784	(0.515, 1.194)
*N*		117	176			
Median (interquartile range)		2.00 (0.00–6.00)	1.00 (0.00–5.00)			
Range		0.00–57.00	0.00–55.00			
Mean ± SD		5.22 ± 9.34	4.08 ± 7.59			
EuraHS QoL score from 1-year survey (score 0–90)	185			0.047	0.588	(0.346, 0.994)
*N*		80	105			
Median (interquartile range)		2.00 (0.00–7.58)	1.00 (0.00–4.00)			
Range		0.00–48.00	0.00–50.00			
Mean ± SD		5.02 ± 7.44	3.59 ± 6.82			
Patient-reported opioid use in the last 30 days at 30-day follow-up	396			< 0.001	0.311	(0.198, 0.483)
0		100/171 (58.48)	182/225 (80.89)			
1–4		37/171 (21.64)	33/225 (14.67)			
5 or more		34/171 (19.88)	10/225 (4.44)			

*N* is the number of non-missing value

*P*-value, Odds Ratio (OR), and 95% confidence interval (CI) calculated using proportional odds regression model

OR reported as TREPP:Lichtenstein

**Table 3:** Domain specific EuraHS patient-reported outcomes

Outcome	*N*	Lichtenstein	TREPP	OR	95% CI
		(*N* = 451)	(*N* = 451)		
EuraHS QoL pain domain score from baseline survey (score 0–30)	363			1.159	(0.808, 1.662)
*N*		162	201		
Median (interquartile range)		6.00 (2.00–11.00)	6.00 (2.00–12.00)		
Range		0.00–28.00	0.00–27.00		
Mean ± SD		6.93 ± 6.29	7.42 ± 6.26		
EuraHS QoL pain domain score from 30-day survey (score 0–30)	493			0.622	(0.453, 0.852)
*N*		235	258		
Median (interquartile range)		3.00 (0.00–6.00)	2.00 (0.00–4.00)		
Range		0.00–24.00	0.00–19.00		
Mean ± SD		4.29 ± 4.87	2.96 ± 3.73		
EuraHS QoL pain domain score from 6-month survey (score 0–30)	293			0.672	(0.413, 1.095)
*N*		117	176		
Median (interquartile range)		0.00 (0.00–2.00)	0.00 (0.00–1.00)		
Range		0.00–20.00	0.00–14.00		
Mean ± SD		1.74 ± 3.67	1.12 ± 2.54		
EuraHS QoL pain domain score from 1-year survey (score 0–30)	185			0.54	(0.296, 0.98)
*N*		80	105		
Median (interquartile range)		0.00 (0.00–2.58)	0.00 (0.00–1.00)		
Range		0.00–17.00	0.00–15.00		
Mean ± SD		1.76 ± 3.01	1.19 ± 2.62		
EuraHS QoL restriction domain score from baseline survey (score 0–40)	358			1.115	(0.776, 1.602)
*N*		160	198		
Median (interquartile range)		10.00 (1.42–20.00)	10.33 (2.00–22.00)		
Range		0.00–40.00	0.00–40.00		
Mean ± SD		11.99 ± 10.94	12.88 ± 11.91		
EuraHS QoL restriction domain score from 30-day survey (score 0–40)	477			0.628	(0.452, 0.871)
*N*		226	251		
Median (interquartile range)		6.00 (0.00–17.03)	2.00 (0.00–10.00)		
Range		0.00–37.00	0.00–38.00		
Mean ± SD		9.19 ± 10.15	6.38 ± 9.08		
EuraHS QoL restriction domain score from 6-month survey (score 0–40)	291			0.526	(0.311, 0.886)
*N*		117	174		
Median (interquartile range)		0.00 (0.00–2.00)	0.00 (0.00–0.00)		
Range		0.00–34.00	0.00–40.00		
Mean ± SD		1.86 ± 5.01	1.34 ± 4.31		
EuraHS QoL restriction domain score from 1-year survey (score 0–40)	183			0.877	(0.447, 1.731)
*N*		78	105		
Median (interquartile range)		0.00 (0.00–1.39)	0.00 (0.00–0.00)		
Range		0.00–21.00	0.00–26.00		
Mean ± SD		1.41 ± 3.49	1.37 ± 3.64		
EuraHS QoL esthetical domain score from baseline survey (score 0–20)	363			1.036	(0.722, 1.487)
*N*		162	201		
Median (interquartile range)		7.00 (3.00–10.00)	8.00 (2.00–10.00)		
Range		0.00–20.00	0.00–20.00		
Mean ± SD		7.30 ± 5.22	7.34 ± 5.23		
EuraHS QoL esthetical domain score from 30-day survey (score 0–20)	493			0.524	(0.381, 0.718)
*N*		235	258		
Median (interquartile range)		4.00 (0.00–8.00)	2.00 (0.00–5.00)		
Range		0.00–20.00	0.00–19.00		
Mean ± SD		4.71 ± 4.47	3.27 ± 4.09		
EuraHS QoL esthetical domain score from 6-month survey (score 0–20)	293			0.926	(0.595, 1.446)
*N*		117	176		
Median (interquartile range)		0.00 (0.00–2.00)	0.00 (0.00–2.00)		
Range		0.00–11.00	0.00–18.00		
Mean ± SD		1.62 ± 2.58	1.61 ± 2.87		
EuraHS QoL esthetical domain score from 1-year survey (score 0–20)	185			0.519	(0.293, 0.916)
*N*		80	105		
Median (interquartile range)		0.00 (0.00–3.00)	0.00 (0.00–1.33)		
Range		0.00–10.00	0.00–10.00		
Mean ± SD		1.81 ± 2.68	1.03 ± 1.98		

*N* is the number of non-missing value

*P*-value, Odds Ratio (OR), and 95% confidence interval (CI) calculated using proportional odds regression model

OR reported as TREPP:Lichtenstein

Additionally, patient-reported opioid use at 30-day follow-up was significantly lower in the TREPP cohort (OR 0.31 [0.20, 0.48]; *p* < 0.001). Although the PSM analysis adjusted for preoperative opioid use, it is important to note opioid prescriptions are likely also influenced by general prescribing patterns of the surgeon. Nonetheless, at 30-day follow-up, matched analyses demonstrated that among those undergoing TREPP repair, 80.9% of patients did not require opioids postoperatively, compared to 58.5% of those who underwent a Lichtenstein repair (Table 2).

### Secondary outcomes

#### Clinical recurrence and perioperative aspects

At 6 months, 1/424 composite recurrences were reported in the TREPP group and 1/428 composite recurrences reported in the Lichtenstein group. The Kaplan–Meier time-to-event log-rank test did not reveal a statistically significant difference in hernia recurrence risk between the TREPP and Lichtenstein repair cohorts (*p* = 0.26).

Lastly, we examined aspects associated with the intra- and post-operative patient care to assess for any pertinent differences among participants undergoing a TREPP or Lichtenstein hernia repair. The 30-day frequency of surgical-site occurrences (SSOs) was 4.3% (17/394) for the Lichtenstein repair cohort compared to 1% (4/401) in the TREPP cohort (OR 0.22 [0.06–0.61]; *p* = 0.007). The majority of SSOs in all groups were seromas. There were no statistically significant differences in 30-day SSOs or surgical-site infections requiring procedural intervention, rates of postoperative bleeding, peripheral nerve injury, postoperative respiratory failure, pulmonary embolism, ileus, bowel obstruction, DVT, or UTI between the TREPP and Lichtenstein repair cohorts (Table 4).

**Table 4:** 30-day clinical outcomes between the Lichtenstein/anterior and OPP/TREPP IH cohorts after propensity score matching

Outcome	*N*	Lichtenstein	TREPP	*P*-value	OR	95% CI
		(*N* = 451)	(*N* = 451)			
30-day surgical-site infection (SSI): Yes	795	0/394 (0.00)	0/401 (0.00)	–	–	–
30-day surgical-site occurrence (SSO-EI): Yes	795	17/394 (4.31)	4/401 (1.00)	0.007	0.223	(0.064, 0.61)
Wound cellulitis: Yes	21	1/17 (5.88)	0/4 (0.00)			
Wound serous drainage: Yes	21	1/17 (5.88)	0/4 (0.00)			
Seroma: Yes	21	11/17 (64.71)	3/4 (75.00)			
Hematoma: Yes	21	3/17 (17.65)	1/4 (25.00)			
Unspecified: Yes	21	1/17 (5.88)	0/4 (0.00)			
30-day any NSQIP complications: Yes	795	2/394 (0.51)	4/401 (1.00)	0.434	1.975	(0.383, 14.304)
Ileus: Yes	795	0/394 (0.00)	0/401 (0.00)			
Bowel obstruction: Yes	795	0/394 (0.00)	0/401 (0.00)			
Pain: Yes	795	0/394 (0.00)	0/401 (0.00)			
PE: Yes	795	0/394 (0.00)	0/401 (0.00)			
Stroke: Yes	795	0/394 (0.00)	0/401 (0.00)			
DVT: Yes	795	0/394 (0.00)	0/401 (0.00)			
Sepsis: Yes	795	0/394 (0.00)	0/401 (0.00)			
Septic shock: Yes	795	0/394 (0.00)	0/401 (0.00)			
MI: Yes	795	0/394 (0.00)	0/401 (0.00)			
Cardiac arrest: Yes	795	0/394 (0.00)	0/401 (0.00)			
UTI: Yes	795	0/394 (0.00)	0/401 (0.00)			
Renal insufficiency: Yes	795	0/394 (0.00)	0/401 (0.00)			
Renal failure: Yes	795	0/394 (0.00)	0/401 (0.00)			
Pneumonia: Yes	795	0/394 (0.00)	0/401 (0.00)			
Postoperative respiratory failure requiring endotracheal intubation: Yes	795	0/394 (0.00)	0/401 (0.00)			
Ventilator > 48 h: Yes	795	0/394 (0.00)	0/401 (0.00)			
Coma > 24 h: Yes	795	0/394 (0.00)	0/401 (0.00)			
Peripheral nerve injury: Yes	795	0/394 (0.00)	0/401 (0.00)			
Post-op bleeding transfusion: Yes	795	0/394 (0.00)	0/401 (0.00)			
Graft/prosthesis/flap failure: Yes	795	0/394 (0.00)	0/401 (0.00)			
Other NSQIP complication: Yes	795	1/394 (0.25)	1/401 (0.25)			
Urinary retention requiring catheter placement: Yes	6	1/2 (50.00)	2/4 (50.00)			

*N* is the number of non-missing value

*P*-value, Odds Ratio (OR), and 95% confidence interval (CI) calculated using proportional odds regression model

OR reported as TREPP:Lichtenstein

## Discussion

Many patients undergo hernia repair at least partly because a hernia affects their QoL. With risk of incarceration and strangulation being relatively low, hernia repair can be seen primarily as a surgical solution that helps patients regain their QoL. As such, surgical repair must optimize patient QoL as well as provide durable and safe outcomes. However, the ideal hernia repair has remained elusive with studies suggesting significant advantages associated with local (over general) anesthesia and posterior mesh placement. The only approaches combining these factors and not violating both anterior and posterior planes are OPP, TREPP, and Kugel repairs. Increasing attention has been directed at assessing QoL after hernia repair in addition to more traditional outcomes like complications, including recurrence and chronic pain. In our recent study comparing TREPP to MIS robotic and laparoscopic approaches, potential benefit in short-term QoL was identified for those individuals undergoing open posterior approaches, potentially due to the generous use of local anesthesia and avoidance of general anesthesia [19]. In this work, we compared two open IHR approaches – TREPP versus Lichtenstein, and found that when confounding variables are accounted for, posterior mesh placement is associated with lower patient-reported postoperative pain, lower use of opioids, and clinically significant difference in return to activity without increasing postoperative complications and risk of recurrence.

Overall median QoL scores at 30-day and 1-year time points were found to be significantly better for those undergoing TREPP compared to Lichtenstein (Table 2). Recent work has sought to establish a minimal clinically important difference (MCID) based on patient-reported QoL scores after an IHR [32, 33]. It has been suggested that the overall MCID for EuraHS-QoL is 10, with domain-specific MCIDs being 3 for pain, 5 for restriction of activities, and 2 for the cosmesis domain [33]. Our data demonstrated a difference of 7 points in the median total EuraHS. While this did not meet the threshold of MCID of 10, when applied to a large population, this difference can be clinically meaningful to a large proportion of patients. There are statistically significant domain-specific differences that can help guide the decision making of what surgery to offer a patient, when multiple procedures are available. For instance, the median QoL scores at 30 days were lower in the TREPP cohort by 1 point in the pain domain 0.622 (95% CI: (0.453, 0.852)), nearly 4 points in the restriction domain (OR 0.63 (95% CI: 0.45, 0.87)), and 2 points in the cosmesis domain (OR 0.524 (95% CI: 0.381, 0.718)). Additional literature is required in order to parse out what is a clinically significant QoL difference for patients. Further, post-operative restriction of mobility is also often guided by the surgeon’s recommendation. While the suggestion of avoiding heavy lifting for 2–4 weeks after surgery is universal, it is certainly possible that differences in specific surgical recommendations not captured in our data might have affected our results.

Prior work comparing an open preperitoneal repair with a Lichtenstein procedure has found that the former is associated with less chronic pain [34, 35]. We also found that significantly less post-operative pain, and consequently lower opioid use, was reported in our TREPP cohort 30 days after surgery. A 70% lower opioid consumption in the TREPP cohort suggests that this approach might benefit from wider adoption from a public health perspective. While we cannot rule out the possibility that at least part of the difference in opioid prescription post-operatively may be practice-dependent or driven by ACHQC initiatives [36], 1.5% of patients develop new persistent opioid use after an IHR [37]. An extrapolation of our findings implies that TREPP can substantially reduce that risk.

The existing literature also supports the notion that an open preperitoneal repair tends to have lower hernia recurrence rate compared to a Lichtenstein repair [38, 39]. Our current analysis found no significant differences in the hernia recurrence risk between the two techniques using time-to-event analysis for up to 5 years after an IHR. Nonetheless, among perioperative outcomes, there was a significantly higher rate of seroma in the Lichtenstein cohort (2.8% versus < 1% in TREPP). We hypothesize that this difference occurs as a result of imbricating transversalis fascia in larger direct hernias, thus eliminating the potential space for serous fluid to accumulate. Importantly, the urinary retention rates were not statistically different between the two cohorts, and neither of the two groups had any reported UTIs in the matched analysis. This finding is not unexpected given the large percentage of patients that avoided general anesthesia and reversal agents. Not only is local anesthesia recommended by international guidelines for open repair of reducible hernias [1], but also it has been shown by several studies to be superior to general and regional anesthesia in terms of postoperative complications, reduced costs, early discharge, reduced pain and patient satisfaction [40–42].

Our findings are in line with previous literature showing that posterior mesh placement through MIS approaches lead to less pain, faster recovery, less restriction on activity and lower opioid use than anterior approaches. For instance, analysis of the French Club Hernia Registry found that preperitoneal repair techniques are associated with significantly lower rates of chronic postoperative inguinal pain, even when examining laparoscopic hernia repair approaches [43]. A TREPP combines all the benefits of an MIS repair with avoidance of GA, which can only be accomplished through an open repair. Thus, our findings contribute to the preponderance of evidence in the literature that a posterior mesh repair, when possible, leads to better outcomes than anterior repair. This is in accordance with existing studies that have found that TREPP/OPP results in similar complication rates compared to a Lichtenstein approach [44], but can result in earlier return to work and improved QoL [45].

It is also worth noting that despite best efforts to account for covariates, our study might be affected by selection biases. Nationally, TREPP repairs are performed by a significantly smaller subset of surgeons (*N* = 7) compared to Lichtenstein IHRs (*N* = 145). As a result, the outcomes measured by the ACHQC are prone to being influenced by the heterogeneity in surgeon training as well as the case volume. While a large number of TREPP cases are being performed by a small subset of high-volume surgeons, in our experience the same surgeons also perform a moderate to large proportion of Lichtenstein IHR cases, which can serve as a theoretical internal control. Altogether, our analysis supports the notion that at least in the appropriate clinical setting and with a skilled surgeon, TREPP represents an important IHR technique, which can offer distinct advantages over a conventional anterior repair.

## Conclusion

Collectively, our data show that an open posterior mesh IHR (TREPP/OPP) is associated with better patient-reported QoL and lower opioid use compared to an anterior mesh IHR (Lichtenstein). These insights call for additional studies and more data collection over time in order to better understand these differences, especially to better estimate the recurrence-free probability after TREPP/OPP compared to the Lichtenstein approach. Nonetheless, since the open posterior mesh repair offers all the benefits of MIS (laparoscopic/robotic), while avoiding general anesthesia, the surgical community should consider further training in this approach as it can be a viable alternative to the traditional open repairs.

## Data Availability

The data used to conduct this analysis and support the results of this paper are directly available from the ACHQC. Data requests can be made directly to the organization at: https://achqc.org/data.
